# Chemical Signaling and Metabolomic Crosstalk in Endophytic Fungi–Medicinal Plant Symbioses for Natural Product Discovery and Sustainable Bioproduction

**DOI:** 10.3390/metabo16030164

**Published:** 2026-02-28

**Authors:** Zhuo Chen, Shilong Jiang

**Affiliations:** College of Pharmacy, Guiyang Kangyang University, Guiyang 550081, China; jhdszmn04@163.com

**Keywords:** endophytic fungi, medicinal plants, chemical signaling, biosynthetic gene clusters (BGCs), spatial metabolomics, sustainable bioproduction

## Abstract

**Background:** Medicinal plants function as complex holobionts, with their therapeutic potential significantly shaped by the associated microbiome, particularly endophytic fungi. These symbionts engage in a sophisticated “chemical signaling” with their hosts, acting as biotic elicitors that modulate plant secondary metabolism while simultaneously responding to host cues to activate their own cryptic biosynthetic gene clusters (BGCs). This review aims to critically summarize the multi-layered mechanisms driving this metabolic crosstalk and evaluate strategies to harness this symbiotic intelligence for natural product discovery. **Methods:** A systematic literature survey spanning the last decade was conducted across major databases. The search specifically targeted studies investigating endophytic fungi in medicinal plants, focusing on experimental designs for BGC activation, applications of spatial metabolomics (matrix-assisted laser desorption/ionization mass spectrometry imaging, MALDI-MSI), and the structural elucidation of novel bioactive natural products through co-culture or in planta models. **Results:** Our analysis reveals that host-derived chemical cues, such as specific root exudates and oxylipins, act as primary triggers to awaken silent fungal BGCs. We collated numerous recently discovered bioactive metabolites—including novel polyketides, highly rearranged terpenoids, and unique alkaloids—demonstrating their potent antimicrobial and cytotoxic properties. Furthermore, a critical evaluation of spatial metabolomics studies demonstrates that metabolic exchange is highly localized at the plant–fungus interface, providing contextual insights that traditional bulk tissue extraction fails to capture. **Conclusions:** This review bridges the gap between ecological understanding and synthetic biology applications. We conclude that translating the mechanisms of this “chemical signaling” into biotechnological strategies offers a sustainable pathway for the bioproduction of high-value pharmaceuticals, thereby reducing reliance on the wild harvesting of medicinal plants.

## 1. Introduction

Medicinal plants remain indispensable for modern drug discovery, yet sustainable supply is increasingly compromised by overharvesting, habitat loss, and inherently low metabolite yields [1,2]. Endophytic fungi have emerged as a promising alternative source of pharmaceutically relevant compounds [3,4]. Since the landmark discovery of paclitaxel-producing Taxus endophytes, it has become evident that these symbionts can independently synthesize “plant” metabolites, offering a strategic solution to relieve pressure on wild resources [2,4].

While endophytic fungi possess the genetic capacity to synthesize diverse bioactive scaffolds—including polyketides, terpenoids, alkaloids, and peptides—genomic analyses reveal that a substantial fraction of their biosynthetic gene clusters (BGCs) remain silent under standard laboratory conditions [3,5]. This suggests that their full metabolic potential is currently underutilized [2,6]. Unlocking this cryptic diversity requires decoding the intimate molecular dialogue of the plant–endophyte holobiont, where reciprocal signaling governs host stress tolerance and regulates fungal BGC expression [7,8].

Recent advances in multi-omics and biotechnology have transformed our ability to harness this symbiosis. Genomics and transcriptomics now enable systematic BGC mining, while mass spectrometry (MS)-based metabolomics facilitates the discovery of novel compounds by comparing axenic and symbiotic states [2,6,9]. Coupled with genome editing and synthetic biology, these technologies pave the way for developing endophytic fungi as scalable “micro-factories” for the sustainable production of high-value pharmaceuticals [2,7,9].

To provide a comprehensive and navigable overview, this review is structured as follows: Section 2 outlines advanced metabolomic strategies (from axenic to spatial in planta models) for deciphering symbiotic interactions. Section 3 details the regulatory networks governing fungal BGCs and strategies for their activation. Section 4 highlights key pharmacologically active natural products discovered through these interactions. Finally, Section 5 synthesizes the mechanistic flow of chemical signaling, while Section 6 discusses translational challenges and applications for sustainable bioproduction.

## 2. Metabolomic Strategies for Deciphering Endophyte–Medicinal Plant Interactions

Metabolomics provides a definitive functional readout of the holobiont by capturing real-time changes in primary and specialized metabolism. Unlike genomics or transcriptomics, which predict potential functions, metabolomics directly links chemical phenotypes to endophyte colonization, stress responses, and therapeutic traits [10,11,12,13]. Untargeted MS-based workflows, particularly when coupled with molecular networking, enable system-level mapping of metabolic shifts under axenic, co-culture, and in planta conditions. These approaches are pivotal for revealing the activation of silent biosynthetic pathways and the emergence of novel metabolite families [14,15,16,17,18]. Global profiling can be further complemented by targeted assays to quantify specific defense metabolites and biomarkers of symbiosis [19,20,21]. Integrating these chemical datasets with ecological models is increasingly revealing how specific compound classes—such as glucosinolates and polyketides—drive endophytic community assembly and host reprogramming [1,21].

### 2.1. Untargeted Metabolomics to Profile Global Metabolic Crosstalk

Untargeted metabolomics is the primary strategy for exploring the largely uncharacterized chemical space of medicinal plant–endophyte interactions. By avoiding a priori selection, high-resolution LC–MS/MS and NMR platforms allow unbiased comparisons between non-inoculated and inoculated hosts, as well as axenic versus co-culture systems [14,15,19]. This comparative approach disentangles host-derived molecules from microbial products, uncovering metabolic rewiring associated with growth promotion and the elicitation of pharmaceutically relevant compounds [11,12,13,22].

Recent applications demonstrate the power of this workflow. For instance, UHPLC-HRMS analysis of medicinal plant cell suspensions has shown that endophyte inoculation stimulates the accumulation of previously undetected phenolic and naphthoquinone derivatives [19,23]. Furthermore, molecular networking has proven essential for visualizing endophyte-specific metabolite clusters that emerge only during host interaction, indicating the awakening of silent gene clusters [15,16,17,18]. In a study of horseradish roots, untargeted LC-MS/MS combined with amplicon sequencing revealed that variation in specific metabolite classes correlated significantly with fungal community structure, underscoring the role of plant chemistry in filtering the microbiome [21]. Similarly, NMR-based profiling of Combretum lanceolatum identified endophyte-induced shifts in primary metabolism that channeled carbon fluxes toward defense-related precursors [20]. A comparison of these fundamental metabolomic experimental designs, highlighting their biological contexts and primary applications, is summarized in Table 1.

### 2.2. Targeted Metabolomics for Pharmacologically Relevant Metabolites

While untargeted workflows drive discovery, targeted metabolomics provides the quantitative rigor necessary to validate pharmacologically relevant compounds. By employing optimized multiple reaction monitoring transitions and authentic standards, this approach offers high sensitivity and absolute quantification, enabling researchers to rigorously link endophyte colonization regimes or culture conditions with the yields of specific alkaloids, terpenoids, and phenolics [13,24,25]. This quantitative precision is essential for both mechanistic studies and the bioprocess optimization of endophyte-based production [24,26,27].

The evolution of “widely targeted” metabolomics now allows for the high-throughput quantification of hundreds of known metabolites, offering a scalable template for quality control. For instance, such platforms have successfully mapped tissue-specific accumulation patterns in medicinal plants like *Taraxacum kok-saghyz*, providing a roadmap for detecting similar metabolic shifts in endophyte-colonized tissues [25,28].

In the specific context of the holobiont, targeted strategies serve critical functions: confirming endophyte-derived analogues of plant drugs, monitoring toxicity markers, and validating bioactives prioritized by untargeted screens [13,25,29,30]. A notable application involved *Artemisia annua* endophytes, where multivariate analysis prioritized quinone derivatives, followed by targeted isolation that identified emodin and physcion as potent antimalarial agents with sub-micromolar IC_50_ values [31]. Furthermore, targeted profiling can decode the pharmacodynamics of herbal medicines by quantifying endogenous metabolite shifts post-treatment [26,29,32]. As analytical libraries expand, these strategies will be pivotal for standardizing endophyte-based phytopharmaceuticals and translating ecological interactions into quantifiable pharmacological outputs [27,29,32].

### 2.3. Spatial Metabolomics to Map Metabolite Distribution Within Host Tissues

Spatial metabolomics, particularly Matrix-Assisted Laser Desorption/Ionization Mass Spectrometry Imaging (MALDI-MSI), bridges the gap between chemical profiling and histology by visualizing in situ metabolite distributions at micrometer resolution [31,33,34]. Unlike bulk extraction, MSI reconstructs ion maps to reveal the precise accumulation of lipids, specialized metabolites, and phytoalexins within tissues. Recent advances have pushed spatial resolution to sub-cellular levels (<5 μm) and introduced multimodal workflows—such as coupling MSI with fluorescence in situ hybridization—to enable the precise assignment of metabolites to specific microbial cells or host structures [33,34,35,36]. It is important to note that while MSI has been extensively validated in clearly defined plant–pathogen models like visualizing fungal virulence factors during infection, its application in mutualistic plant–endophyte symbioses is a rapidly emerging frontier. In endophyte studies, researchers must carefully design MSI workflows to differentiate defensive chemical warfare from cooperative, symbiotic chemical exchanges.

In the context of the holobiont, MSI is pivotal for distinguishing host- versus microbe-associated metabolic territories. Studies on maize, grapevine, and rice have successfully differentiated pathogen-derived toxins from localized host defense “hotspots” that bulk analysis fails to resolve [37,38,39]. Beyond mapping, MSI supports functional target validation; for instance, visualizing the disruption of fungal melanin pathways in *Magnaporthe*-infected barley directly linked metabolic inhibition to reduced pathogenicity [40]. These spatially resolved signatures provide a mechanistic layer complementary to bulk metabolomics [41,42].

**Figure 1:**
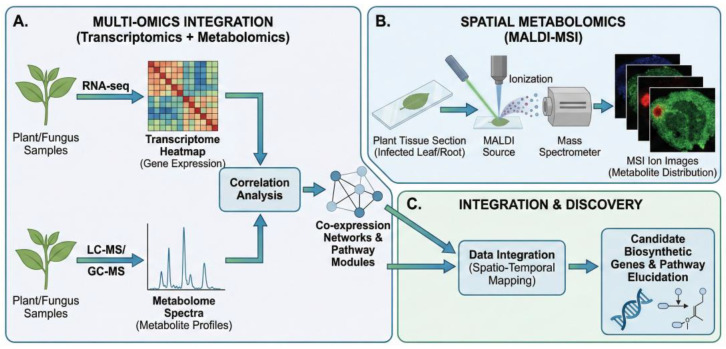
Integrated multi-omics and spatial metabolomics workflows for deciphering metabolic interactions: (**A**) Multi-omics integration workflow combining transcriptomic (RNA-seq) and metabolomic (LC-MS/GC-MS) profiles through correlation analysis to generate co-expression networks and pathway modules; (**B**) Spatial metabolomics utilizing MALDI-MSI for the in situ visualization of metabolite distribution within plant tissue sections; (**C**) Integration and discovery phase, where the combination of multi-omics networks and spatial data enables spatio-temporal mapping, leading to the precise localization of metabolites and elucidation of candidate biosynthetic pathways. Arrows indicate the direction of the workflow and data integration steps. Different colors are used to visually distinguish between the transcriptomic, metabolomic, and spatial imaging modules.

Despite these capabilities, challenges remain. A persistent trade-off exists between spatial resolution and sensitivity, often limiting the detection of low-abundance specialized metabolites even with advanced ionization technologies [43]. Furthermore, sample preparation poses a major bottleneck; the high water content of plant tissues can cause metabolite delocalization during sectioning, necessitating standardized protocols to minimize artifacts at the delicate root–microbe interface [44,45]. As summarized in Figure 1, integrating this high-resolution spatial imaging (MALDI-MSI) with transcriptomics–metabolomics correlation analysis provides a comprehensive workflow: it enables the precise localization of metabolites and the identification of their biosynthetic pathways in situ, thereby deciphering the spatiotemporal logic of the holobiont.

### 2.4. Integrative Multi-Omics for Pathway Elucidation

Integrative multi-omics combines genomic, transcriptomic, and metabolomic data to reconstruct the causal networks driving host–endophyte interactions. By analyzing these layers jointly, researchers can move beyond descriptive gene lists to identify key regulatory nodes [11,46]. For instance, co-expression networks in *Camellia sinensis* and barley have successfully linked MAPK signaling cascades to defense-related metabolic modules, while metabolite-GWAS in fungal pathogens has mapped regulatory polymorphisms to virulence factors [47,48,49,50,51].

Deciphering these complex traits requires a robust framework where high-quality genome assemblies for BGC annotation are integrated with time-resolved dual RNA-seq to capture dynamic transcriptional modules. This transcriptomic landscape is then coupled with matched metabolomics profiling, specifically targeting central carbon flux and specialized defense compounds. Subsequent network-based integration—such as overlaying weighted gene co-expression networks with metabolite abundance—can prioritize candidate enzymes for functional validation [11,46,52].

However, bottlenecks persist. First, the immense chemical diversity of the holobiont limits metabolome coverage, often leading to systematic false negatives [13,53]. Second, a distinct “annotation gap” remains, as only a small fraction of detected features can be confidently assigned due to incomplete spectral libraries [44,54]. Finally, data integration across disparate spatial scales (single-cell MSI vs. bulk RNA-seq) poses significant computational challenges for robust network analysis [55,56].

### 2.5. Bioinformatic Tools and Workflows for Endophyte–Host Metabolomic Data

Processing dual-organism datasets requires robust bioinformatic workflows capable of disentangling complex host–microbe signals. Complete pipelines typically progress through three stages: automated pre-processing, statistical functional analysis, and integrative pathway reconstruction.

**Preprocessing and Molecular Networking.** For untargeted LC–MS/MS data, automated pipelines such as UmetaFlow and MetMiner now provide end-to-end solutions for feature detection and alignment, offering direct connectivity to Global Natural Products Social Molecular Networking (GNPS) [57,58,59]. This integration is critical for Molecular Networking, which clusters structurally related metabolites to visually distinguish endophyte-specific analogs from the host background. For instance, in *Astrocaryum*–endophyte systems, such workflows successfully separated host- and microbe-enriched metabolite classes, enabling the tracing of distinct chemical lineages within the holobiont [17,57]. For NMR-based profiling, similar multivariate workflows facilitate binning and spectral database matching to track primary metabolic shifts during colonization [22].

**Statistical and Functional Interpretation.** Downstream analysis is increasingly centralized in web-based suites like MetaboAnalyst 4.0 and XCMS Online. Beyond standard univariate/multivariate statistics, these platforms offer modules for Joint Pathway Analysis, allowing researchers to overlay genomic or proteomic data directly onto metabolomic dysregulation maps [60,61]. These systems-biology capabilities are essential for linking specific metabolic features—such as upregulated defense compounds—to phenotypic traits like stress tolerance or disease resistance.

**Pathway Reconstruction and Integration.** To move from correlation to causation, specialized tools are emerging to model metabolic handoffs. MEANtools employs reaction-rule-based algorithms to link mass features with transcriptomic data, reconstructing candidate biosynthetic pathways de novo [62]. In parallel, genome-scale pipelines like METABOLIC profile functional networks from metagenomic data to predict metabolite exchange potential [62]. Coupling these inferred networks with measured metabolomic changes provides a coherent framework to decipher the “chemical language” of the plant–endophyte dialogue.

While these computational tools have democratized metabolomics, selecting the appropriate pipeline remains critical for plant–endophyte studies. Molecular networking is exceptionally powerful for clustering structurally related analogs and identifying known compound families through spectral library matching, but it often struggles with the ‘dark matter’ of entirely novel scaffolds characteristic of endophytic fungi. In contrast, fragmentation tree-based methods excel at predicting molecular formulas and substructures for unknown metabolites ab initio, independent of spectral databases. Rather than applying bioinformatic tools indiscriminately, researchers should follow a logical decision tree: utilizing GNPS primarily for the rapid dereplication of known scaffolds, while reserving targeted MS/MS molecular networking and in silico BGC prediction tools like antiSMASH for prioritizing novel or highly divergent BGC products. Therefore, a hybrid workflow—combining GNPS for rapid dereplication of host plant metabolites with SIRIUS/CANOPUS for the structural elucidation of novel fungal natural products—represents the current gold standard for dissecting the complex chemical interplay in these symbiotic systems.

**Mechanistic Summary of Metabolomic Strategies.** Moving from basic axenic screening to integrative multi-omics and MSI provides a progressive workflow. Untargeted metabolomics generates the initial holistic profiles, multi-omics links these expressed profiles to their underlying genetic capacity (BGCs), and spatial metabolomics visually maps their ecological reality in situ.

## 3. BGCs and Regulatory Networks in Endophytic Fungi

Endophytic fungi harbor a vast reservoir of BGCs that encode core scaffold-forming enzymes—such as nonribosomal peptide synthetases, polyketide synthases (PKSs), and terpene synthases—alongside necessary tailoring enzymes and transporters [51,63]. Large-scale genome mining has revealed hundreds of thousands of such BGCs, the vast majority of which remain uncharacterized, underscoring the enormous cryptic chemodiversity within the endosphere [64]. However, most clusters are transcriptionally silent under standard laboratory conditions. Their activation is governed by complex regulatory networks involving pathway-specific transcription factors, global regulators, chromatin remodeling, and environmental signals [63,64,65,66].

Recent insights from model filamentous fungi illustrate the regulatory architectures expected in endophytes. Gene co-expression networks have successfully predicted trans-acting transcription factors that control multiple BGCs, including those lacking obvious internal regulators [51,63]. Experimental overexpression of these factors often modulates the production of multiple secondary metabolites, revealing extensive connectivity between cluster-bound and genome-wide regulation [51,63,65]. Furthermore, global regulators such as the velvet family have been shown to coordinately influence conidiation, stress responses, and polyketide biosynthesis, thereby broadly reshaping the secondary metabolite profile [66]. These layers intersect with epigenetic mechanisms, where histone modifications determine chromatin accessibility; targeted perturbation of this machinery can effectively activate silent BGCs and diversify metabolite output [67]. Evolutionary analyses further suggest that BGC regulation is highly plastic, with transcription factors frequently divergent or repurposed to control different clusters in related species [68].

### 3.1. Diversity and Classification of BGCs in Endophytes of Medicinal Plants

Endophytes of medicinal plants possess a diverse biosynthetic repertoire that mirrors and often extends that of free-living fungi. Genome mining of individual isolates typically reveals dozens of clusters per genome, dominated by Type I polyketide synthase (T1 PKS), Non-Ribosomal Peptide Synthetases (NRPS), terpene, and hybrid PKS–NRPS systems [69,70,71]. For instance, a single *Talaromyces* sp. from *Syzygium samarangense* encodes 76 predicted BGCs, including rare phosphonate and β-lactone clusters [69], while a *Helotiales* endophyte from Bergenia contains over 77 clusters spanning PKS, NRPS, and ribosomally synthesized peptide (RiPP) families [71].

Classifying these BGCs reveals distinct enzymatic signatures shaped by host association. PKS clusters frequently utilize reducing and non-reducing T1 PKSs to generate scaffolds such as anthraquinones; in *Hypericum*-derived endophytes, specific non-reducing PKS clusters have been linked to hypericin-like biosynthesis [70]. NRPS and hybrid PKS–NRPS clusters contribute substantially to peptide-based diversity, driving the production of diketopiperazines and complex hybrid scaffolds across fungal lineages [64,72]. Terpene and meroterpenoid clusters are also widespread, often embedding terpene synthase genes that yield plant-associated volatiles like linalool or utilizing unusual prenyltransferase–cyclase logic to generate polyketide–terpenoid hybrids [71,73]. While large-scale genomics suggests that most BGCs fall into taxonomically restricted gene cluster families [74,75], endophytes appear to assemble these core modules into unique repertoires, such as the distinct anthraquinone-linked clusters found in *Hypericum* symbionts versus the meroterpenoid-enriched profiles of *Bergenia* endophytes [70,71].

### 3.2. Genome Mining and in Silico Prediction of Bioactive Pathways

Systematic genome mining has revolutionized the discovery of latent biosynthetic capacity in medicinal plant endophytes. At the genomic level, computational platforms such as antiSMASH now enable the rapid identification and rule-based annotation of BGCs, providing cluster-level comparisons to experimentally characterized pathways [76].

Recent applications illustrate the power of this approach. Whole-genome sequencing of an endophytic *Talaromyces* sp. from *Syzygium samarangense* revealed 76 BGCs—spanning polyketide, nonribosomal peptide, and terpene families—and allowed the specific assignment of clusters to scaffolds like squalestatin S and fusarin [69]. Similarly, analysis of the mangrove endophyte *Fusarium multiceps* identified 33 BGCs, 23 of which lacked homology to known pathways, highlighting the immense reservoir of cryptic chemistry in these symbionts [77].

Beyond detection, computational frameworks are increasingly capable of inferring structure and function. Comparative analysis of over 1000 fungal genomes has organized thousands of predicted BGCs into gene cluster families, creating an interpreted atlas to facilitate dereplication [78]. Furthermore, machine-learning models trained on BGC–metabolite pairs can now predict antibacterial or cytotoxic activities directly from sequence data with promising accuracy [79]. In medicinal plant research, such targeted mining has successfully pinpointed non-reducing polyketide clusters in *Hypericum* endophytes as putative sources of hypericin-like anticancer metabolites [70].

### 3.3. Regulatory Circuits and CRISPR-Based Activation of BGCs

Secondary metabolite biosynthesis in endophytes is governed by a multilayered hierarchy involving pathway-specific transcription factors, global regulators like the velvet family, and epigenetic chromatin remodeling [80,81,82]. Because many BGCs remain silent under standard axenic conditions, advanced molecular toolkits are required to unlock their potential. CRISPR–Cas technologies, particularly transcriptional activation (CRISPRa) and precise genome editing, offer robust methods to awaken these silent clusters. By deleting global negative regulators, replacing native promoters, or modifying epigenetic landscapes, researchers can systematically trigger secondary metabolite production and capture large BGCs for heterologous expression, bypassing the limitations of traditional culture modifications [83,84,85]. The mapping of these distinct regulatory layers to their corresponding targeted activation strategies is systematically summarized in Table 2.

### 3.4. Challenges in Mimicking the Host Microenvironment

A fundamental bottleneck in endophyte research is the discrepancy between the nutrient-rich, homogeneous conditions of axenic culture and the resource-limited, chemically complex environment of the host tissue. Standard media often favor fast-growing copiotrophs, failing to recover metabolically specialized endophytes or to elicit the expression of host-dependent BGCs [86].

**Simulating the Endosphere.** Recent “environmental simulation” strategies attempt to bridge this gap. Leaf-based culture pads, created by overlaying agar on processed leaf tissue, significantly improved the recovery of recalcitrant microbiota compared to standard R2A medium [87]. Similarly, seed-mimicking media tailored to the nutritional profile of germinating soybean revealed compartment-specific functional traits relevant to salt stress [86]. However, these ex vivo systems still lack active transport and dynamic immune responses.

**The In Vitro–In Planta Gap.** Functional discrepancies remain a challenge; dual-culture screens often yield “false positives”—strains that inhibit pathogens in vitro but fail to colonize or protect the host in planta due to ecological constraints [88]. Infection models in *Echinacea* further highlight that colonization outcomes are strictly dictated by host genotype and tissue identity [89]. Moving forward, truly predictive platforms will require organotypic cultures and microfluidic “root-on-a-chip” devices that emulate the spatiotemporal gradients and signaling complexity of the natural endosphere [86,88,89].

### 3.5. Integrating Ecological and Genetic Data into Unified Models

Predicting the behavior of plant–endophyte systems requires models that fuse ecological context with host–microbe genotypes. Constraint-based metabolic modeling, particularly Genome-Scale Models (GEMs), serves as a critical bridge by embedding genomic data into a stoichiometric framework [90,91]. For example, simulations using bacterial GEMs have successfully predicted phyllosphere community assembly with >89% accuracy, demonstrating how metabolic cross-feeding structures microbial niches [91].

Future unified models are moving toward systematic multi-omics integration. By coupling plant multiscale models (genome-to-phenome) with microbial GEMs, researchers can link gene regulatory networks to whole-plant physiology and ecological outcomes [90,92,93]. Achieving this vision depends on combining mechanistic reconstructions with machine learning and adhering to FAIR data standards to ensure robust, iterative model refinement [94,95,96].

### 3.6. Linking BGCs to Metabolites Through Combined Omics and Metabolomics

Connecting orphan BGCs to their products is the primary bottleneck in natural product discovery. Correlation-based metabologenomics has emerged as a leading solution, successfully pairing BGCs with metabolites by statistically analyzing their co-occurrence across large strain collections [97,98].

Complementary strategies leverage chemical similarity metrics and machine learning to predict BGC–metabolite links even in smaller datasets [79,99]. Validation relies on robust heterologous expression platforms or condition-dependent multi-omics, which have successfully elucidated dozens of cryptic pathways [100,101]. This integrated pipeline—from genome mining to experimental validation—is essential for decoding the complex chemodiversity of the endosphere.

## 4. Pharmacologically Active Natural Products from Endophytic Fungi of Medicinal Plants

Endophytic fungi colonizing medicinal plants have emerged as a renewable reservoir of pharmacologically active natural products that mirror, complement, or significantly extend the chemical repertoire of their hosts. Numerous strains isolated from traditional medicinal species produce structurally diverse secondary metabolites—including polyketides, terpenoids, alkaloids, xanthones, and peptides—with activities spanning antimicrobial, anticancer, antioxidant, antiplasmodial, and anti-inflammatory effects [102,103,104].

**Chemical Mimicry and Alternative Supply.** A distinct advantage of these symbionts is their ability to synthesize specific “phytochemicals” originally attributed solely to the host plant. This capacity provides a potential sustainable production platform for scarce drugs, circumventing limitations such as low accumulation levels and the overharvesting of endangered species [105]. Parallel bioprospecting efforts in ethnomedicinal floras, such as those in Nigeria and Asia, continue to uncover endophytic extracts with potent bioactivities against pathogens and oxidative stress-related targets, confirming that only a small fraction of this host-associated diversity has been tapped [104,106].

**Novel Scaffolds and Therapeutic Leads.** Beyond reproducing host-like metabolites, endophytes generate novel scaffolds not detected in plant tissues. Chromatographic and GC–MS profiling has revealed unique nitrogen-containing heterocycles, phenolic derivatives, and unusual lipids that exhibit strong antibacterial and cytotoxic properties [102,104]. Importantly, many of these compounds demonstrate selectivity indices compatible with early-stage drug discovery, distinguishing them from general toxins [104]. Selected examples of these pharmacologically active natural products discovered between 2020 and 2025 are summarized in Table S1.

The growing body of pharmacological characterization supports endophytic fungi as a complementary reservoir of lead compounds that align with traditional ethnobotanical uses while expanding the accessible chemical space [102,103]. The following subsections will detail these major activity classes, exploring structure–activity relationships and the challenges involved in translating these endophyte-derived hits into preclinical development.

### 4.1. Structural Diversity and Therapeutic Potentials of Endophyte-Derived Metabolites

Endophytic fungi produce a vast array of structurally diverse, pharmacologically active secondary metabolites, including complex polyketides, highly rearranged terpenoids, non-ribosomal peptides, and unique alkaloids [102,107,108,109,110]. Rather than serving merely as ecological defense molecules, these compounds frequently exhibit target-specific bioactivities ranging from broad-spectrum antimicrobial to neuroprotective and anticancer properties (generalized findings and specific examples are extensively detailed in Table S1). The structural plasticity of these metabolites—driven by unique cyclization enzymes and hybrid modular systems like PKS–NRPS—demonstrates that specific scaffold variations and functional group modifications dictate therapeutic selectivity. This positions endophyte-derived scaffolds as versatile, tunable leads for pharmaceutical development, effectively expanding the accessible chemical space beyond host-plant synthesis.

### 4.2. Case Studies: Endophyte-Derived Analogs of Host-Plant Marker Compounds

A growing body of work demonstrates that endophytes can biosynthesize the same or closely related analogs of signature (“marker”) metabolites that define the chemical identity and quality of their host plants. In some cases, the endophyte produces the authentic plant marker; in others, it generates structurally modified congeners or enhances the accumulation of host-derived scaffolds, blurring the boundary between plant and microbial chemotypes [2].

The phenomenon of chemical mimicry, where endophytes produce “plant-exclusive” metabolites, has sparked intense debate regarding its evolutionary origins. Two competing hypotheses dominate the field: Horizontal Gene Transfer and Convergent Evolution. The Horizontal Gene Transfer hypothesis posits that fungi acquired biosynthetic genes directly from their host plants during long-term co-evolution; however, genomic analyses of taxol-producing fungi have largely failed to identify plant-homologous gene sequences, weakening this argument. Conversely, the Convergent Evolution hypothesis suggests that fungi and plants independently evolved distinct enzymatic pathways to assemble identical scaffolds under similar selection pressures. Current genomic evidence increasingly favors the latter, indicating that endophytes have evolved unique, structurally distinct enzymes that functionally mimic plant biosynthetic steps to produce compounds like camptothecin and vinblastine.

**Schitriterpenoids and Related Triterpenes in *Kadsura angustifolia***. The medicinal liana *Kadsura angustifolia* is characterized by highly oxygenated schitriterpenoids, such as nigranoic acid, which serve as key quality markers. The endophytic fungus *Umbelopsis dimorpha* SWUKD3.1410, isolated from this host, was fermented on substrates containing host-plant material. Comparative analysis revealed that the fungus could produce the authentic marker nigranoic acid alongside 11 other schitriterpenoids, including novel highly oxygenated analogs not previously reported from the plant [111]. These findings indicate that *U. dimorpha* can both mimic host biosynthesis and drive diversification through unique biotransformation pathways [111].

**Classical Anticancer “Plant Drugs” Re-assigned to Endophytes.** Several hallmark anticancer natural products historically regarded as plant-exclusive have been traced to endophytes. For instance, high-resolution MALDI-MSI of *Putterlickia* roots revealed that maytansine, a potent cytotoxic agent, is actually produced by cortical bacteria rather than the plant itself [13]. Similarly, the macrocyclic astin peptides, originally attributed to *Aster tataricus*, were found to be synthesized by the fungal symbiont *Cyanodermella asteris* [13]. These cases confirm that the “marker” compounds defining the pharmacological value of these species are largely of endophytic origin.

**Endophyte-Induced Analogs of Host Flavonoids and Chromones.** Endophytes can also be induced to generate “marker-like” analogs through metabolic crosstalk. The fungus *Botryosphaeria ramosa* L29, when cultured with a host-derived flavanone precursor, produced a series of novel chromone derivatives that structurally resemble the host’s phenylpropanoid markers [3]. These induced metabolites exhibited antifungal activities against *Fusarium oxysporum* comparable to commercial fungicides, illustrating how host precursors can steer fungal biosynthesis toward bioactive, near-isosteric analogs [3].

**Implications for Marker-Based Standardization and Bioproduction.** Collectively, these findings complicate the traditional standardization of herbal medicines but simultaneously open new avenues for bioproduction. Since endophytes can produce authentic markers like maytansine and astins, novel analogs like *Kadsura* triterpenoids, or precursor-derived congeners like *Botryosphaeria* chromones, they represent scalable “micro-factories” for high-value therapeutics that serve as sustainable alternatives to harvesting endangered plant biomass [3,4,111].

### 4.3. Structure–Activity Relationships and Lead Optimization Perspectives

Available data on endophyte-derived metabolites from recent studies reveal clear structure–activity relationships that are critical for guiding rational optimization toward drug-like leads. Beyond simple potency screenings, these investigations highlight how specific scaffold variations, absolute configurations, and functional group modifications dictate target selectivity and pharmacological safety.

**Stereochemistry and Scaffold Selectivity.** Precise stereochemical control is often a determinant of bioactivity. For instance, in polyketides isolated from the *Huperzia serrata* endophyte *Phaeosphaeria* sp. LF5, the specific furanone scaffold of aspilactonol I conferred potent acetylcholinesterase inhibitory activity (IC_50_ 6.26 μM), whereas the related isocoumarin derivative was threefold less potent (IC_50_ 21.18 μM) [112]. This suggests that the stereochemical arrangement of the lactone ring is critical for binding affinity in neurodegenerative targets. Similarly, among alkaloids from *Melia* endophytes, the β-carboline skeleton demonstrated a superior Selectivity Index (SI > 16.1) against Influenza A (H1N1) compared to other nitrogenous congeners, highlighting how scaffold rigidity can decouple antiviral efficacy from host cytotoxicity [113].

**Functional Group Tuning and Activity Decoupling.** Subtle modifications in oxidation state and substitution patterns can dramatically alter therapeutic profiles. In the study of *Alternaria* sp. MG1, specific polyketide analogues exhibited potent inhibitory activity against cyclooxygenase-2 (COX-2), while structurally related congeners in the same extract lacked this anti-inflammatory potential, pinpointing specific oxygenation patterns required for enzyme engagement [114]. Furthermore, novel triterpenoids from *Penicillium ochrochloron* displayed moderate cytotoxicity (IC_50_ 6.5–17.8 μM) but retained strong radical-scavenging and antifungal activities [115]. This separation of activities suggests that the cycloartane core can be chemically tuned—likely through side-chain modification—to enhance antimicrobial specificity while reducing off-target toxicity to human cells [115].

**From Culture Optimization to In Silico Design.** Lead optimization also extends to biosynthetic regulation and virtual screening. For cytotoxic metabolites from marine-derived *Aspergillus* sp., optimizing physicochemical culture parameters significantly enhanced the yield of bioactive quinoline and indole scaffolds, improving the crude extract’s IC_50_ against HeLa cells to 24 ± 2 μg/mL [116]. Looking forward, integrated platforms are increasingly combining synthetic biology with computational tools. Recent work on terpene cyclases has demonstrated that rational enzyme engineering can drive the diversification of meroterpenoid skeletons [108], while in silico molecular docking now routinely filters endophyte metabolites against emerging targets, such as the main protease (3CLpro) of SARS-CoV-2, to prioritize high-affinity hits for synthesis [117].

## 5. Chemical Signaling and Symbiotic Interplay in Endophyte–Host Systems

Endophyte–host associations are now defined as chemically driven symbioses, where mutual recognition, colonization, and long-term compatibility are orchestrated by a complex network of signaling molecules, phytohormones, and secondary metabolites. Far from being passive inhabitants, endophytes actively perceive host-derived cues—such as root exudates and defense compounds—and reprogram their own metabolism to establish symptomless colonization while modulating host physiology and stress resilience [118].

This “chemical signaling” involves deep metabolic rewiring that extends to the broader microbiome. Multi-omics studies reveal that secondary metabolites act as central mediators of these tripartite interactions. For instance, in *Epichloë*–grass systems, fungal colonization alters root exudate profiles, which subsequently restructures bacterial communities (Proteobacteria and Firmicutes) under varying nitrogen regimes [119]. Similarly, comparative metabolomics in long-lived hosts like the palm *Astrocaryum sciophilum* and tropical gymnosperms demonstrates that specific fungal lineages drive significant variation in the host leaf metabolome, supporting a model of finely tuned, lineage-dependent metabolic interplay [17,120].

To persist in chemically defended niches, endophytes deploy specialized adaptations. A *Fusarium solani* endophyte from *Camptotheca acuminata* possesses amino-acid substitutions in topoisomerase I that confer resistance to the host’s cytotoxic alkaloid camptothecin, facilitating coexistence and potentially convergent biosynthesis [118]. Furthermore, endophytic consortia can collectively upregulate plant pathways; in *Papaver somniferum*, distinct community members complement biosynthetic steps to modulate morphine and noscapine production [118]. These findings position the holobiont as a chemically integrated unit where reciprocal signaling underpins both ecological fitness and pharmaceutical yield.

### 5.1. Signaling Molecules Mediating Colonization and Niche Establishment

Successful colonization is a multi-step process beginning with chemotactic sensing and culminating in intracellular accommodation. Root exudates rich in amino acids and organic acids serve as primary attractants, guiding rhizosphere microbes to the root surface [121]. While well-characterized in nitrogen-fixing symbioses where Nod factors initiate infection [11], similar molecular decoding of complex exudate blends allows non-nodulating endophytes to locate suitable entry sites [121,122].

**Phytohormone Crosstalk.** Once attached, hormonal signaling determines ingress. The endophyte *Acremonium* sp. D212 secretes indole-3-acetic acid and jasmonic acid (JA) to manipulate host immunity; colonization is severely compromised in rice mutants defective in JA perception (coi1-18) or auxin signaling (miR393b), but can be restored by exogenous methyl jasmonate [123]. In contrast, the beneficial endophyte *Azoarcus olearius* elicits a JA-dependent response in rice roots that limits endophyte density without triggering the salicylic acid (SA) defense pathways typically activated by pathogens like *Xanthomonas*, illustrating a selective “gating” mechanism via hormone signaling [124].

**Effectors as Molecular Tools.** Endophytes also secrete specific effectors to secure their niche. Transcriptomics of *Serendipita* root endophytes revealed the upregulation of hundreds of effector genes during colonization, including a GH18-CBM5 chitinase that suppresses competing phytopathogenic fungi while protecting the host [125]. In intracellular associations, such as *Methylobacterium* in Scots pine, genomic analysis identified Type IV secretion systems and eukaryote-like ankyrin repeats likely used to modulate host nuclear processes [126]. Thus, effector repertoires function not just as virulence factors, but as essential communication signals for symbiotic stability.

Crucially, these host-derived signaling molecules function not merely as ecological cues but as direct transcriptional triggers for fungal secondary metabolism. Recent mechanistic studies suggest that plant phytohormones, such as SA and jasmonates, can cross the fungal cell wall and interact with specific fungal G-protein coupled receptors or intracellular sensory proteins. This interaction often initiates a phosphorylation cascade that subsequently activates pathway-specific regulatory genes located within cryptic BGCs. Consequently, the “chemical signaling” effectively converts an ecological signal into a genetic response, breaking the chromatin silencing of BGCs that remain dormant under axenic laboratory conditions.

### 5.2. Modulation of Host Primary Metabolism and Specialized Metabolism

Endophytic colonization triggers a rapid reprogramming of host metabolism, acting as a “metabolic gear shift” that reallocates carbon and nitrogen from primary maintenance to enhanced specialized metabolite biosynthesis.

**Primary Metabolism Reprogramming.** Endophytes strategically adjust central carbon flux to prime chemical defenses. In *Atractylodes lancea,* protein elicitors from *Pseudomonas fluorescens* increase photosynthetic carbon input, directly fueling the production of sesquiterpenoids like atractylone [118]. Similarly, metabolomic analysis of *Combretum lanceolatum* colonized by *Diaporthe phaseolorum* revealed elevated levels of threonine and malic acid—precursors essential for downstream defense compounds [22]. This coordinated remodeling extends to lipid and sugar metabolism, as seen in *Astragalus mongholicus*, where the endophyte *Talaromyces coprophilus* reshapes glycerophospholipid and ubiquinone pathways to support a new metabolic state [127].

**Redirecting Flux to Specialized Pathways.** Beyond precursor supply, endophytes exert profound control over secondary metabolic networks. In *Echinacea purpurea*, bacterial endophytes upregulate valine decarboxylase, boosting the formation of immunomodulatory alkamides [118]. Elicitors can also enforce branch-specific channeling; in *Salvia miltiorrhiza* hairy roots, polysaccharide elicitors significantly increased tanshinone I and salvianolic acid B levels while modulating rosmarinic acid pools, consistent with differential flux routing through specific CYP450-dependent branches [128]. At the community scale, correlations between endophytic diversity and gentiopicroside levels in *Gentiana* spp. confirm that these lineages jointly engineer the host’s medicinal chemical space [129].

### 5.3. Endophyte-Mediated Enhancement of Plant Stress Tolerance and Defense

Endophytic microbes serve as internal stress modulators, enhancing host resilience to abiotic and biotic challenges by coordinating osmotic adjustment, antioxidant defense, and immune signaling.

**Abiotic Stress Resilience.** Endophytes mitigate osmotic and oxidative damage through systemic physiological adjustments. Salt-tolerant bacteria in *Arachis hypogaea* significantly improve growth under high salinity (200 mM NaCl) by boosting antioxidant enzyme activities (SOD, CAT) and inducing genes linked to lipid metabolism and ion channels [130]. Similarly, the fungus *Fusarium equiseti* confers drought tolerance in maize by maintaining relative water content and upregulating photosynthesis and terpene biosynthesis genes, effectively coupling osmotic balance with chemical defense [131].

**Biotic Defense and Crosstalk.** In parallel, endophytes reinforce resistance against pathogens. *Trichoderma* spp. trigger Induced Systemic Resistance via jasmonate/ethylene signaling and WRKY53-dependent expression of PR1, providing broad-spectrum protection [132]. Endophytic actinomycetes further modulate phenylpropanoid metabolism and pathogenesis-related proteins to improve fitness under disease pressure [133]. Crucially, these defense layers are often integrated; for instance, salt-tolerant peanut endophytes simultaneously enhance antioxidant capacity and secondary metabolite biosynthesis, illustrating how endophytes deploy a dual-function shield against environmental and biological stressors [130,132].

### 5.4. Co-Metabolism, Metabolic Channeling, and Shared Pathways

**Figure 2:**
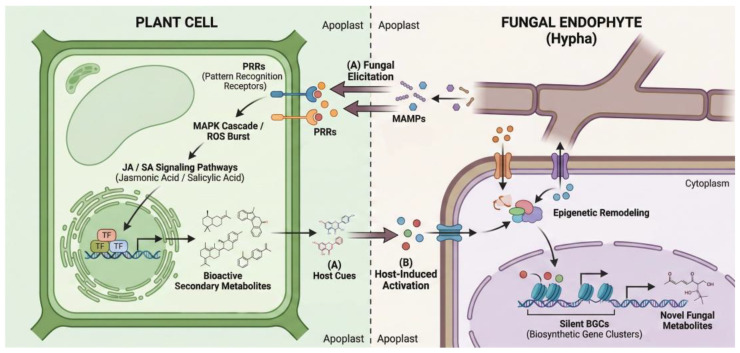
Chemical signaling at the Plant–Endophyte Interface: (**A**) Fungal elicitors (Microbe-Associated Molecular Patterns, MAMPs) trigger host immune signaling (MAPK, JA/SA pathways) to upregulate bioactive secondary metabolites; (**B**) Host-derived chemical cues induce epigenetic remodeling in fungi, awakening silent biosynthetic gene clusters (BGCs). Arrows indicate the direction of signal transduction and molecular interactions. Different colors distinguish the plant and fungal endophyte compartments.

Endophyte–host associations are increasingly recognized as metabolically integrated systems where partners utilize, modify, or jointly produce small molecules within partially overlapping networks. This phenomenon of co-metabolism is exemplified by the symbiosis between the grass *Achnatherum inebrians* and the clavicipitaceous endophyte *Epichloë gansuensis*. Under ammonium supply, integrated microbiome–metabolome analysis revealed that nitrogen-responsive pathways—specifically cysteine, methionine, and tyrosine metabolism—function as shared “currencies” bridging the host and its microbiome. As illustrated in Figure 2, this bidirectional exchange involves the flow of host-derived primary metabolites to the endophyte, which in turn supplies bioactive secondary metabolites to the plant, facilitating a visible “chemical signaling” that underpins the holobiont’s stability. Network analysis further indicated a mechanism of metabolic channeling, where flux is routed through a limited set of shared intermediates that act as hubs connecting plant roots to specific bacterial taxa, while other metabolites like L-DOPA show partner-specific routing [119].

However, the extent of this sharing varies by system and metabolite class. Comprehensive profiling of the Amazonian palm *Astrocaryum sciophilum* and its foliar endophytes suggests a model of selective compartmentalization. While central primary metabolites (fatty acids, amino acids, carbohydrates) form a jointly accessible pool, the vast majority of specialized metabolites remain distinct to either the host or the fungus, allowing for chemical differentiation within the holobiont [17].

Beyond passive sharing, endophytes can actively drive the accumulation of biosynthetic precursors for both partners. In *Combretum lanceolatum,* colonization by *Diaporthe phaseolorum* significantly elevates levels of threonine, malic acid, and N-acetyl-mannosamine. These primary metabolites feed directly into downstream defense pathways and simultaneously serve as carbon/nitrogen sources for the fungus, illustrating a reciprocal strategy to fuel specialized metabolism [22]. Collectively, these studies define a spectrum of interaction—from tight metabolic channeling to selective precursor provisioning—that underpins the stability and chemical complexity of endophyte–host holobionts [17,22,119].

### 5.5. Ecological and Evolutionary Drivers of Metabolomic Diversification

Metabolomic diversification in plant–endophyte systems is shaped by interacting ecological filters and evolutionary processes across multiple scales.

**Community and Intraspecific Drivers.** At the community level, chemical dissimilarity among tree species is a strong predictor of biodiversity, with environmental factors like climate promoting secondary metabolite divergence [134]. Similarly, elevational gradients can select for distinct phytochemical profiles within populations of grasses like *Festuca rubra*, driving localized chemical endemism [135].

**Organ-Specific and Microbiome-Mediated Variation.** Plant organs impose distinct selection regimes; fruits consistently exhibit higher chemical richness and structural complexity than leaves or roots, supporting organ-specific diversification roles [136,137]. Crucially, the microbiome adds another axis of variation. In *Echium vulgare*, root metabolomes covary with developmental stage and specific prokaryotic assemblages, while global multi-omics data show that microbial community composition is tightly linked to metabolite turnover across habitats [138,139]. Evolutionary analysis of *Xylariales* genomes further reveals that ecological generalism selects for “metabolic hyperdiversity” with generalist endophytes possessing significantly more BGCs than their specialized relatives [140].

### 5.6. Mechanisms of Host Specificity and Co-Evolution

Patterns of host specificity in endophytes are well-documented, yet the underlying mechanisms remain complex. Field surveys indicate that host plant identity is the primary determinant of endophyte community structure, often overriding environmental factors [141,142].

**Metabolic Filtering and Co-adaptation.** Host-driven metabolic filtering is a key mechanism; distinct root exudate profiles selectively recruit specific microbiota like *Burkholderia* [143]. Evidence for tighter co-adaptation comes from vertically transmitted symbionts. Cophylogenetic analysis of *Epichloë* and pooid grasses reveals significant host–endophyte co-divergence, where the endophyte enhances host reproductive traits in a population-specific manner [144,145].

**Genomic Plasticity.** Rapid evolution of interaction traits facilitates this specialization. In pathogenic systems, accessory chromosomes and epigenetic variation enable fast adaptation to host resistance [146]. Similar mechanisms likely operate in endophytes, where strain-level host preferences and distinct effector repertoires suggest that genomic plasticity plays a central role in defining host range and lifestyle [146,147]. Disentangling whether this specificity arises from immune gating, metabolic choice, or co-evolving signaling networks remains a central challenge [143].

**Mechanistic Summary: Plant-Driven vs. Endophyte-Driven Metabolic Changes.** To fully decipher the chemical signaling within the holobiont, it is essential to distinguish the directional flow of metabolites. Plant-driven changes typically initiate the dialogue: roots secrete primary metabolites for chemotaxis and specific secondary cues that act as keys to unlock silent fungal BGCs. Conversely, endophyte-driven changes manifest as the release of novel fungal polyketides and non-ribosomal peptides that not only inhibit local competitors but also act as elicitors themselves, systemically upregulating the host plant’s own defensive pathways such as jasmonic acid and salicylic acid signaling. This bidirectional crosstalk ultimately results in the enhanced accumulation of active medicinal components in the host tissue.

## 6. Translational Applications and Sustainable Bioproduction

Plant–endophyte metabolomics is driving a shift toward lower-impact agricultural and pharmaceutical production systems. Numerous endophytes isolated from medicinal plants synthesize high-value bioactives—such as taxanes and cyclic peptides—that were once thought to be plant-exclusive. This discovery offers a sustainable alternative to the destructive harvesting of endangered species, enabling the production of these compounds via controlled fermentation [148,149].

Significant strides have been made in optimizing these microbial factories. In *Fusarium* cultures, tuning medium composition and parameters achieved an order-of-magnitude increase in N-methylsansalvamide yields, reaching titers compatible with industrial development [150]. Similarly, solid-state bioreactors using *Trichoderma asperellum* enabled the co-production of volatile lactones, enzymes, and conidia, demonstrating how endophytes can be integrated into circular, residue-based bioprocesses for crop protection [150].

Despite this promise, axenic cultures often suffer from genomic instability and the attenuation of secondary metabolite production. To overcome these constraints, strategies such as pathway amplification, co-culture fermentation, and epigenetic modification are being deployed to activate cryptic clusters and stabilize yields [149,150]. Looking forward, the integration of omics-guided engineering with microbiome design holds the potential to deliver climate-resilient crops and sustainable therapeutics, anchoring endophyte metabolomics within the broader bio-based economy [1,148,149,150,151,152].

### 6.1. Heterologous Expression of Endophytic Fungal BGCs

Heterologous expression is a central strategy to access cryptic metabolites and develop scalable production systems when native endophytes are uncultivable or genetically intractable. Genomic surveys reveal that the vast majority of endophytic BGCs remain orphan; for instance, only 5 of 47 predicted BGCs in *Neonectria* sp. DH2 were linked to known products, highlighting a vast unexplored chemical space [153].

**Chassis Engineering and Plug-and-Play Platforms.** Fungal Artificial Chromosome -based platforms in *Aspergillus nidulans* have successfully captured and expressed dozens of full-length BGCs, yielding numerous previously undescribed metabolites [100]. Similar “plug-and-play” workflows have been adapted to hosts like *Fusarium graminearum*, where in vivo yeast recombination enabled the production of complex multi-gene pathways with titers comparable to native strains [154].

**Optimized Hosts.** The choice of host is critical. *Aspergillus nidulans* has been engineered as a “clean” chassis by deleting endogenous clusters to reduce background noise [155]. More recently, *Penicillium crustosum* was refactored using CRISPR/Cas9 to improve gene-targeting efficiency and marker flexibility, validating a cross-species host-switching strategy [156]. In parallel, deep metabolic engineering of *Aspergillus oryzae*—modifying flux through the mevalonate and acetyl-CoA pathways—resulted in up to 65-fold increases in terpenoid yields, converting a food-grade mold into a high-performance cell factory [157].

**Yeast-Based Screening.** Complementing filamentous systems, synthetic biology platforms in *Saccharomyces cerevisiae* allow for high-throughput screening of refactored BGCs. While intron processing remains a challenge, strategies combining cDNA reconstruction with standardized promoters are effectively generalizing yeast as a tool for the systematic mining of endophytic natural products [155,158].

However, a major “translational gap” persists between the discovery of bioactive endophytes and their industrial deployment. While wild isolates often produce high-value metabolites in planta or in co-culture, they frequently suffer from rapid diverse attenuation and vanishingly low titers when grown in isolation (axenic fermentation). This physiological collapse occurs because the endophyte relies on the host for specific precursors and signaling cues—a dependency defined as obligate co-metabolism. Consequently, direct fermentation of wild strains is rarely commercially viable. This bottleneck necessitates the transition from simple strain selection to synthetic chassis engineering, where the relevant BGCs are captured and refactored into robust, industrially domesticated hosts that are independent of plant signals.

### 6.2. Synthetic Biology and Chassis Engineering for Scalable Production

Synthetic biology serves as the bridge between discovering endophyte-derived potential and realizing industrial-scale bioproduction. By leveraging modular genetic toolkits and design–build–test cycles, researchers are optimizing fungal and yeast chassis for enhanced stability and yield.

**Modular Toolkits and Chassis Domestication.** For filamentous fungi, standardized transcription units assembled via Golden-Gate strategies enable the rapid generation of strain libraries with altered growth and secretion profiles [159]. Platforms like DIVERSIFY systematically screen *Aspergillus* species to identify optimal backgrounds for specific pathways, effectively de-risking scale-up decisions [159]. In parallel, advances in *Pichia pastoris* utilizing CRISPR/Cas systems have raised gene disruption efficiencies to ~80%, facilitating the multiplex integration of heterologous genes into food-grade hosts [160].

**Metabolic Rewiring for High Flux.** Rational engineering increasingly combines global metabolic rewiring with fine-tuned regulation. In *Saccharomyces cerevisiae*, extensive modification of the mevalonate pathway pushed carbon flux toward sesquiterpene biosynthesis, achieving 875 mg/L of the biofungicide 5-epi-jinkoheremol [161]. Similar strategies—overexpressing precursor nodes and deleting competing sinks—are being generalized to non-conventional yeasts with advantageous traits like thermotolerance [162]. For filamentous fungi, engineering also targets morphology and secretion; modifying cell wall architecture and vesicle trafficking components can significantly boost protein and metabolite export, “moulding the mould” for bioreactor performance [159,163].

### 6.3. Bioprocess Optimization for Industrial Fermentation of High-Value Metabolites

Bioprocess optimization is critical for translating lab-scale successes into robust industrial fermentation, aiming to maximize titer and productivity while minimizing stress-induced variability.

**Media and Feeding Strategies.** Optimizing nutrient ratios is a primary lever for production. In *Yarrowia lipolytica*, co-feeding strategies combined with in situ extraction raised retinol titers to 5.4 g/L, demonstrating how phase-specific feeding balances growth and production [164]. For dual-product systems, pH-controlled fed-batch strategies have enabled high-yield co-production of 3-hydroxypropionic acid and 1,3-propanediol from glycerol [165].

**Data-Driven Control and Scale-Up.** Advanced control systems are transforming scale-up. AI-guided regulation, integrating real-time spectroscopy with neural network models, increased gentamicin C1a titers by over 54% by redirecting flux toward NADPH generation [166]. In gas fermentation, kinetic modeling supported the continuous production of acetone and isopropanol from waste gases at industrial rates, verified as carbon-negative by life-cycle assessment [167]. Even in complex solid-state fermentations, response surface methodology optimized environmental parameters to boost vanillin biosynthesis by nearly 400% [168]. Crucially, MS-based fingerprinting now serves as a metric for scaling natural products, revealing that rigorous process matching is required to preserve metabolite profiles across scales [169].

### 6.4. Endophyte-Based Alternatives to Wild Harvesting of Medicinal Plants

Uncontrolled harvesting for high-value metabolites has pushed species like *Nothapodytes nimmoniana* and *Taxus* spp. toward vulnerability, necessitating host-independent production platforms [170,171]. Endophytic microbes offer two scalable biological alternatives: direct fermentation of the endophyte or endophyte-assisted cultivation of the host plant [170,171,172].

**Direct Fermentation Platforms.** A proof-of-concept for replacing wild harvesting is the microbial production of the anticancer alkaloid camptothecin. Screening of *N. nimmoniana* endophytes identified *Alternaria alstroemeriae* and *A. burnsii* as high-yield producers up to 426.7 µg/g. Crucially, *A. burnsii* maintained production stability over 12 sequential subcultures, overcoming the “yield attenuation” bottleneck common in fungal fermentation. Isotope labeling confirmed de novo biosynthesis, establishing a sustainable production platform independent of endangered biomass [170]. Other systems show similar promise; endophytes from *Ocotea* and *Cynodon* yield potent antibacterial and antioxidant metabolites detectable in axenic culture [173,174].

**Bioinoculants for Cultivated Plants.** Alternatively, endophytes function as “living fertilizers” that enhance the quality of cultivated crops. In *Salvia miltiorrhiza*, inoculation with specific endophytes modulated carbon–nitrogen metabolism and antioxidant status, significantly increasing the accumulation of tanshinones and phenolic acids in sterile seedlings [172]. This approach allows for the design of tailored microbial consortia that maximize pharmacopeial quality in plantation-grown materials, reducing the economic incentive for wild collection [172].

### 6.5. Regulatory, Safety, and Standardization Considerations for Endophyte-Derived Products

The commercialization of endophyte-derived products requires navigating a complex regulatory landscape that overlaps with biostimulants, probiotics, and herbal medicines. Safe deployment relies on three pillars: biosafety of the organism, quality control of the metabolite, and rigorous standardization.

**Biosafety and Environmental Risk.** The release of novel endophytes is evaluated akin to the introduction of new biological agents. New Zealand’s risk-assessment model classifies endophytes into “acceptable” or “unacceptable” categories based on pathogenicity, toxigenicity, and gene transfer potential, regulating them under the Hazardous Substances and New Organisms Act [175]. This structured approach aligns with biosafety frameworks for genetically modified organisms, where case-specific assessment focuses on the interaction between the modified organism and the receiving environment [176].

**Quality Control and Manufacturing Hygiene.** For human use, strict quality controls are essential. Surveys of natural products in Quito revealed that up to 43% of samples exceeded pharmacopoeial limits for aerobic counts, with some containing pathogenic *Pseudomonas*, highlighting critical gaps in manufacturing hygiene [177]. Similarly, commercial probiotics frequently suffer from mislabeling and contamination [178,179,180]. Therefore, endophyte formulations must demonstrate genetic stability, purity, and the absence of adventitious contaminants throughout their shelf life [181].

**Standardization and Legislation.** Regulatory frameworks for biofertilizers and novel foods offer a path forward. In the EU, plant biostimulants must demonstrate efficacy and safety under Regulation (EU) 2019/1009 [182]. Meanwhile, purified endophyte metabolites intended as nutraceuticals fall under “novel food” regulations, requiring comprehensive dossiers on toxicology, allergenicity, and production history [183,184]. Harmonizing these definitions is crucial to mitigate safety risks arising from inadequate testing [177,184].

### 6.6. Limitations and Challenges in Natural Product Discovery from Endophytes

Despite the immense potential of endophytic fungi, translating their symbiotic chemistry into scalable bioproduction faces several critical challenges. First, a significant “unculturable” barrier remains; traditional isolation media often fail to mimic the complex, resource-limited microenvironment of the host tissue, leaving metabolically specialized endophytes unrecovered [86,87]. Second, even when successfully isolated, endophytes frequently suffer from severe yield attenuation or complete silencing of BGCs during axenic fermentation due to the absence of host-derived chemical signals and the disruption of obligate co-metabolism [88]. Furthermore, ensuring the reproducibility of metabolite profiles across different scales—from laboratory flasks to industrial bioreactors—poses substantial bioprocessing hurdles [169]. Overcoming these limitations requires a shift from simple axenic screening toward advanced synthetic biology interventions, organotypic co-culture systems, and the rational engineering of robust industrial chassis.

## 7. Conclusions

Plant–endophyte associations constitute a pervasive yet under-exploited dimension of biology, offering direct pathways to crop resilience and ecosystem sustainability. Experimental evidence confirms that these symbionts modulate phytohormones and mitigate abiotic stresses to deliver measurable yield benefits under field conditions [185]. Concurrently, they serve as potent biocontrol agents and alternative producers of pharmacologically active metabolites, underscoring their dual potential in agriculture and biotechnology [151,185,186].

**From Description to Mechanism.** The integration of omics has shifted the field from descriptive phenomenology to mechanistic understanding. Metagenomics reveals a biosynthetic diversity that far exceeds culture-based recovery [151], while transcriptomics and metabolomics are beginning to map how endophytes reprogram host stress-response networks [11]. Emerging AI-driven frameworks now merge these multi-omics layers to predict complex trait architectures, providing the foundation for unified models that couple ecological context with host–microbe genotypes [187,188].

**Figure 3:**
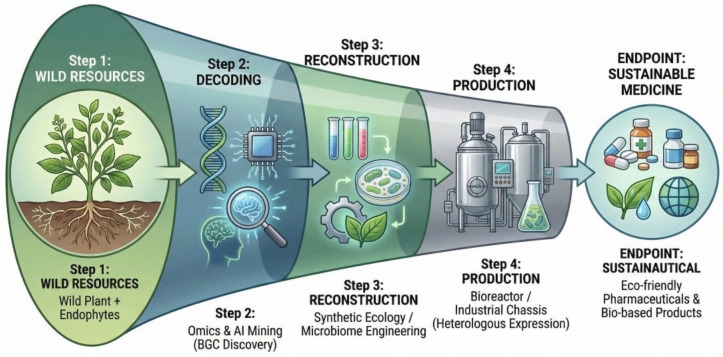
A translational roadmap from ecological insights to sustainable biomanufacturing. Strategies include mining silent BGCs via Artificial Intelligence, designing synthetic microbial consortia, and heterologous expression in industrial chassis to replace wild plant harvesting. Arrows indicate the directional progression of the translational pipeline. Different background colors are used to visually distinguish the distinct stages of the process.

**Future Roadmap.** Realizing the full potential of plant–endophyte systems demands a unified trajectory moving from opportunistic isolation toward precision discovery and robust deployment. This comprehensive trajectory, visualized as a translational roadmap in Figure 3, integrates ecological insights with advanced biomanufacturing strategies to replace unsustainable wild harvesting. Future exploitation must systematically explore under-sampled host lineages and extreme environments using genome-resolved multi-omics and deep learning to link specific BGCs to quantifiable plant functions, rather than relying solely on taxonomic novelty [189,190,191]. Translating such discoveries into reliable products requires re-designing the inoculant pipeline around host–genotype–environment matching, prioritizing indigenous consortia and host-mediated microbiome engineering to ensure stable in situ colonization [192,193,194]. While synthetic biology offers tools for creating endophytes with programmable traits, their deployment hinges on rigorous biosafety safeguards and benchmarking against elite wild isolates [195]. Ultimately, the sustainable scaling of these technologies necessitates standardized efficacy protocols and equitable access to strain repositories, ensuring that precision endophyte discovery contributes to global food security rather than widening regional technological gaps [190].

Finally, the integration of Artificial Intelligence (AI) heralds a new era for endophyte research. The application of protein structure prediction tools, such as AlphaFold2 and RoseTTAFold, is poised to resolve the longstanding bottleneck of “orphan” BGCs. By accurately predicting the 3D structures and substrate pockets of core biosynthetic enzymes, researchers can now infer the likely structure of the final metabolite directly from genomic data, bypassing years of trial-and-error fermentation. Coupled with AI-driven genome mining, this structural insight allows for the in silico prioritization of high-value clusters, shifting the paradigm from random screening to rational, structure-guided discovery of next-generation therapeutics from the plant microbiome.

## Figures and Tables

**Table 1:** Schematic comparison of metabolomic experimental designs for endophyte research.

Experimental Design	Biological Context	Primary Application in Discovery	Limitations
Axenic Culture (Standard)	Fungi grown in isolation ^1^	Baseline profiling; isolation of constitutively expressed metabolites	High rate of silent BGCs; lacks ecological relevance
Co-culture/OSMAC	Fungi grown with elicitors, host extracts, or microbial competitors	Awakening silent BGCs; simulating biotic stress	Artificial environment; may not perfectly mimic in planta conditions
In planta Models	Fungi inoculated into sterile host plants or roots	Mapping true symbiotic chemical signaling and holobiont metabolism	Highly complex matrix; difficult to distinguish host vs. fungal origin

^1^ Standard isolation media typically include Potato Dextrose Agar or Potato Dextrose Broth.

**Table 2:** Mapping regulatory layers to BGC activation strategies.

Regulatory Layer	Biological Mechanism	Targeted Activation Strategy
Global Regulators	Broad pleiotropic control ^1^	CRISPR/Cas9-mediated deletion of repressors or overexpression of activators
Pathway-Specific	Transcription factors situated within or near a specific BGC	Promoter engineering; CRISPRa targeting specific genes
Epigenetic	Chromatin remodeling ^2^	Application of chemical epigenetic modifiers in culture ^3^
Environmental/Ecological	Host signals or microbial competition triggering stress pathways	Co-culture frameworks; addition of biotic/abiotic elicitors mimicking the host

^1^ Examples include LaeA and the Velvet complex. ^2^ Primarily through histone methylation or acetylation. ^3^ Such modifiers include histone deacetylase or DNA methyltransferase inhibitors.

## Data Availability

No new data were created or analyzed in this study. Data sharing is not applicable to this article.

## Supplementary Materials

The following supporting information can be downloaded at: https://www.mdpi.com/article/10.3390/metabo16030164/s1, Table S1: Selected examples of pharmacologically active natural products from endophytic fungi of medicinal plants (2020–2025).

## Abbreviations

The following abbreviations are used in this manuscript:

AI	Artificial Intelligence
BGCs	Biosynthetic Gene Clusters
GEMs	Genome-Scale Models
GNPS	Global Natural Products Social Molecular Networking
JA	Jasmonic Acid
MALDI-MSI	Matrix-Assisted Laser Desorption/Ionization Mass Spectrometry Imaging
MS	Mass Spectrometry
NRPS	Non-Ribosomal Peptide Synthetases
PKS	Polyketide Synthases
SA	Salicylic Acid
